# *Campylocarpon fasciculare* (*Nectriaceae*, *Sordariomycetes*); Novel Emergence of Black-Foot Causing Pathogen on Young Grapevines in China

**DOI:** 10.3390/pathogens10121555

**Published:** 2021-11-29

**Authors:** Pranami D. Abeywickrama, Wei Zhang, Xinghong Li, Ruvishika S. Jayawardena, Kevin D. Hyde, Jiye Yan

**Affiliations:** 1 Beijing Key Laboratory of Environment Friendly Management on Fruit Diseases and Pests in North China, Institute of Plant and Environment Protection, Beijing Academy of Agriculture and Forestry Sciences, Beijing 100097, China; 5971105509@lamduan.mfu.ac.th (P.D.A.); zhwei1125@163.com (W.Z.); lixinghong@ipepbaafs.cn (X.L.); 2 Centre of Excellence in Fungal Research, Mae Fah Luang University, Chiang Rai 57100, Thailand; ruvishika.jay@mfu.ac.th (R.S.J.); kdhyde3@gmail.com (K.D.H.); 3 School of Science, Mae Fah Luang University, Chiang Rai 57100, Thailand; 4 Innovative Institute of Plant Health, Zhongkai University of Agriculture and Engineering, Guangzhou 510225, China

**Keywords:** grapevine black-foot disease, new record, molecular phylogeny, pathogenicity, viticulture

## Abstract

The black-foot disease of grapevine is one of the most destructive diseases in viticulture and it is caused by a complex of soil-borne fungi. This study aimed to identify the species associated with black-foot disease in young grapevines in vineyards of China. Fungal isolates were identified as *Campylocarpon fasciculare*, based on both morphological and multi-locus phylogenetic analysis of ITS, *tef1–α* and *ß-tubulin* sequence data. For the first time in China, we report *Campylocarpon fasciculare* associated with symptomatic young grapevines. Koch’s postulates were performed on *Vitis vinifera* cv. Summer Black (SB) in a greenhouse and to confirm the pathogenicity on grapevines. This work improves the knowledge of black-foot disease in Chinese vineyards and will be helpful to growers in their decisions regarding vinicultural practices, planting and disease management.

## 1. Introduction

Grapes (*Vitis vinifera* L. and/or *Vitis* spp.), are one of the most widely grown fruit crops in the world mainly for fresh fruits and wine production [1]. Due to their adaptability to diverse climatic conditions, viticulture has expanded worldwide. As one of the places of origin for *Vitis* species, China has had a long history concerning both viticulture and brewing wine [2]. Chinese grape and wine industry have grown rapidly along with China’s overall economic growth [2]. China now has the second-largest surface area of vines to produce wine, table grapes or dried grapes. Furthermore, China has its place in world viti-viniculture, being the main table grape producer, the third-largest dried grape producer, the tenth-largest wine producer and the fifth-largest wine consumer [3].

Black-foot disease is one of the most destructive diseases in grapes worldwide, especially in nurseries and young vineyards as this can cause serious economic losses [4,5,6,7,8]. Heavily wet and poorly drained soils increased the severity of black-foot disease and caused significant crop losses in many vineyards [4,5,6,7,8]. This has increased the annual plant loss up to 20–40% in many countries. [4,6,7,8]. Halleen et al. [4] stated that black-foot pathogens such as *Campylocarpon fasciculare* and *C. pseudofasciculare* caused 22.5% and 17.5% vine mortality, respectively, in glasshouse conditions. Large investments are needed to establish and maintain a vineyard. Therefore, the long lifespan of vines is essential for obtaining economic success in vineyards. Due to the black-foot disease, replanting costs have increased causing significant economic losses to the grape industry [4,5,6,7,8].

This disease was first reported in 1961 in France and subsequently in many grape growing regions, including Australia [8], California [6,7,9] Chile [10], Italy [11], Lebanon [12], New Zealand [4], Portugal [13], Spain [14], South Africa and Uruguay [15]. This disease primarily affects young grapevines up to eight years of age and symptoms of the disease include stunted aboveground grapevines showing shortened internodes with chlorotic leaves scorched by water stress [4,7]. Further, small, black, necrotic, sunken lesions on roots and/or dark purplish or reddish-brown discolored wood tissues can be observed starting at the basal end of the rootstock.

Seven fungal genera have been associated with black-foot disease including *Campylocarpon*, *Cylindrocarpon*, *Cylindrocladiella*, *Dactylonectria*, *Ilyonectria*, *Neonectria* and *Thelonectria* [16,17,18]. Previous studies have stated that there are 24 cylindrocarpon-like species associated with the black-foot disease of grape [4,6,8,10,13,17,19,20,21,22]. These pathogens are mostly soil-borne [4,5,17,23,24,25] and can infect roots and stem bases. As these pathogens can survive in soil, they can act as a source of inoculum for the next disease cycle even if infected plants were removed. Inocula can spread throughout the field when infested soil is shifted by water or machinery [4,5]. However, the disease cycle of these causative pathogens in vineyards has not been subjected to detailed studies [26].

Cylindrocarpon-like taxa have undergone extensive taxonomic revisions. With the study by Lombard et al. [17], *Dactylonectria* was established to resolve the paraphyletic nature of *Ilyonectria* [23,24,26]. Recently, a detailed study was carried out by Lawrence et al. [27], to evaluate the taxonomy and phylogenetic placement of cylindrocarpon-like species that are associated with grapevine and other perennial crops (e.g., almond, cherry, kiwi, olive, peach, pistachio and walnut) in California. This study identified 12 cylindrocarpon-like taxa belonging to five genera (*Campylocarpon*, *Dactylonectria*, *Ilyonectria*, *Neonectria* and *Thelonectria*) [27]. Most studies on black-foot disease on grapevines are from Australia, Chile, Italy, Lebanon, New Zealand, Portugal, South Africa, Spain, Uruguay and the United States [21]. There are no records of black-foot disease on grapevines from Chinese vineyards (Table 1).

Thus, this study aimed to determine the occurrence of black-foot disease in a young vineyard in China and to characterize the *Campylocarpon* species associated with this disease utilizing morpho-molecular analyses and pathogenicity studies.

## 2. Results

### 2.1. Disease Symptoms Identified in the Field

**Figure 1:**
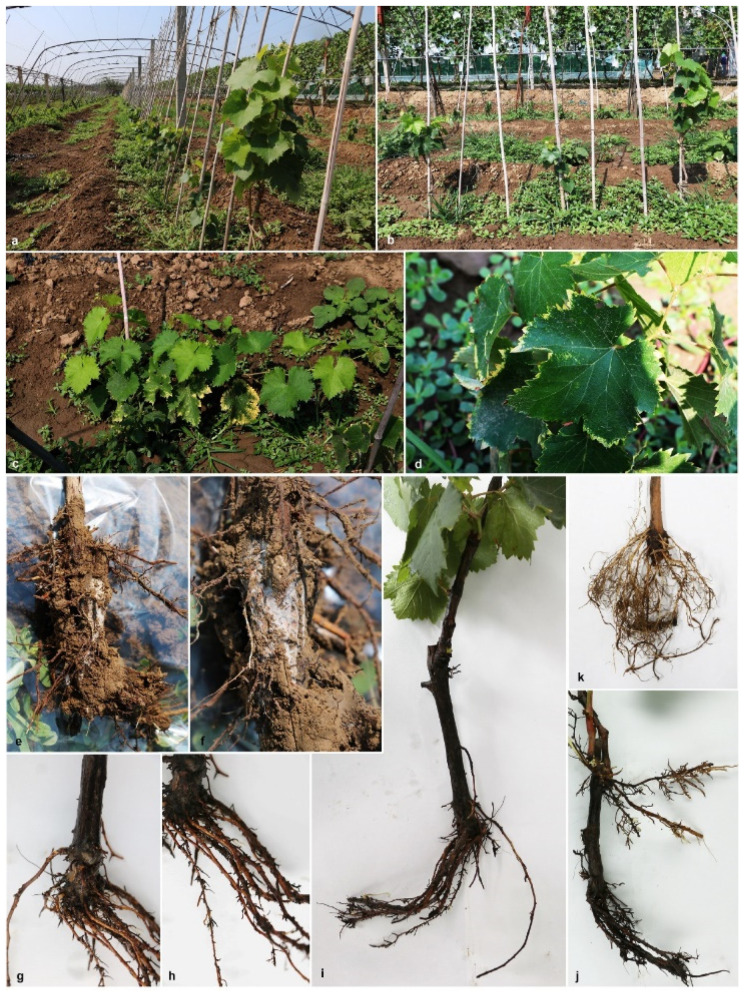
Field symptoms associated with black-foot disease of grapevine in Chinese vineyard. **a**–**c**. Young grapevines showing weak vegetation, stunted growth and reduced vigour. **d**. Leaf chlorosis. **e**,**f**. Mycelial mat covered the root parts of the grapevines. **g**,**h**. Poor root development. **i**. J-rooting. **j**. Poor root development and second layer of roots, growing parallel to the soil surface. **k**. Healthy root system from a healthy grapevine.

**Figure 2:**
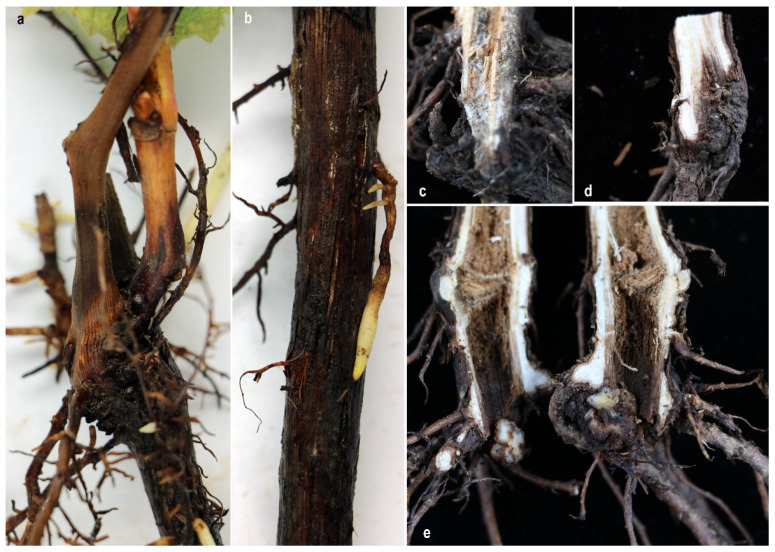
Symptoms associated with black-foot disease of grapevine. **a**. Black discoloration and necrosis of wood tissue which develops from the base of the rootstock. **b**. Black discoloration and necrosis of new roots. **c**. Mycelium covered inside the wood tissues at the base of rootstock. **d**,**e**. Longitudinal sections of a rootstock showing dark-brown to black discoloration.

During the survey of grapevine trunk and root disease causing pathogens in China, symptomatic young rooted grapevines were observed in a vineyard in Beijing province (Figure 1). Different types of external symptoms on the plants were observed such as stunting, vine vigour reduction, shortened internodes, shoot dieback and leaf discoloration with chlorosis (Figure 1). Sunken necrotic symptoms near the basal end of the root stock and reduction of root hairs were also observed on uprooted plants (Figure 1, Figure 2).

### 2.2. Isolation of Fungi

Two isolates of *Campylocarpon*, were obtained from disease affected wood tissues which were at the basal end of the root stock and necrotic roots.

### 2.3. Morphological Characterization

**Figure 3:**
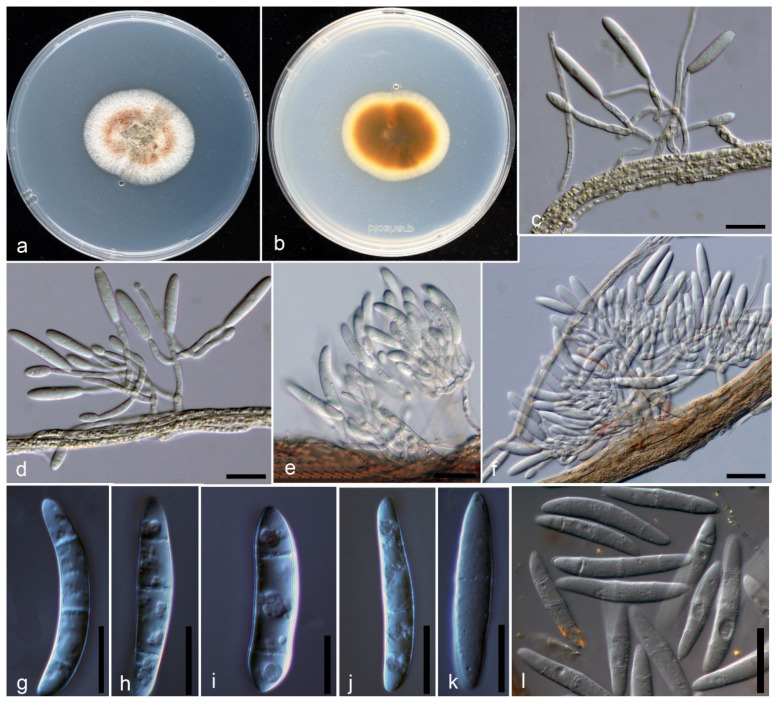
*Campylocarponfasciculare* (JZB 3300001). **a** Colonies on PDA-upper view. **b** Colonies on PDA- reverse view. **c**,**d** Branched conidiophore arising from apical part of hyphal stand. **e**,**f** Fascicles of branched conidiophores arising from brownish hyphal strands. **g–l** Macroconidia. Scalebars: **c**–**l** = 10 µm.

Isolates of the recovered species in this study fit well within the species concept of *Campylocarpon* Both isolates (JZB 3300001, JZB 3300002) demonstrated similar culture characteristics with the ex-type of *C. fasciculare* (CBS 112613) with slight dimensional differences (Figure 3a,b). These differences may have occurred probably due to environmental factors. Colonies on potato dextrose agar (PDA) were slow growing, reaching 2–4 cm in diameter after seven days of incubation at 25 ± 2 °C.

#### Taxonomy

**Figure 4:**
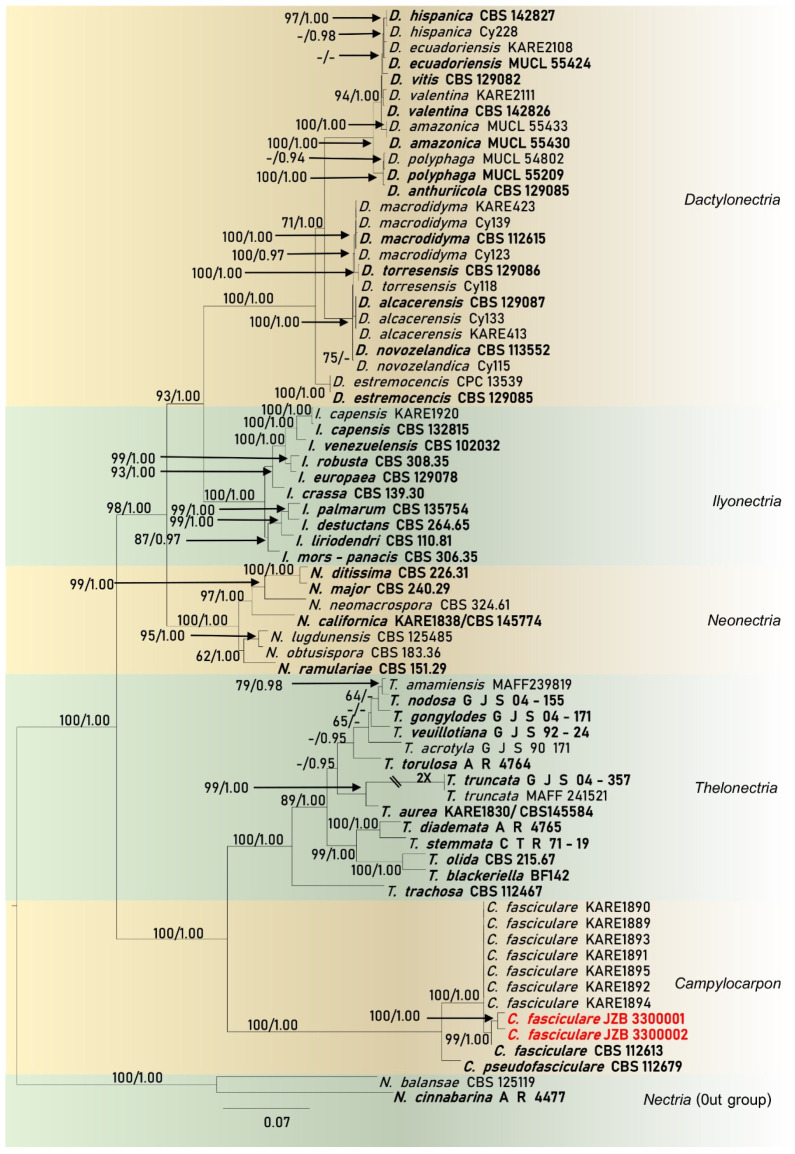
Phylogram generated from maximum likelihood analysis (RAxML) of cylindrocarpon-like fungi based on ITS, *tef1–α* and *ß-tubulin* sequence data. Maximum likelihood (ML) bootstrap values equal to or above 60%, Bayesian posterior probabilities (BYPP) equal to or above 0.90 are given at the nodes. Isolate/specimen number or culture collection number is noted after the species name. The tree is rooted to *Nectria cinnabarina* (A.R. 4477) and *N. balansae* (CBS 125119). The ex-type strains are indicated in bold. The newly generated sequences are indicated in red. Hyphen (-) represents support values below 60% ML and 0.90 BYPP. The scale bar indicates the number of nucleotide changes.

Isolates associated with black-foot of *V. vinifera* cv. SB clustered within the genus *Campylocarpon*, in the multi-locus phylogeny (Figure 4). The species described here, is identified as *Campylocarpon fasciculare* (Figure 3), which was first described as a devastating grapevine black-foot causing pathogen from South Africa in 2000 [4].

***Campylocarpon fasciculare*** Schroers, Halleen and Crous (Figure 3)

Index Fungorum no: IF 500116

Sexual morph: Undetermined. Asexual morph: Culture on PDA, *Conidiophores* (11.9-) 19.1 (–28.7) × (1.9–) 2.9 (–5.5) µm (*n* = 9) initially simple, short, branched, conidiophores consisting of single phialides or terminating in a whorl of phialides. *Phialides* monophialidic, cylindrical, tapering toward the apex. *Macroconidia* on PDA produced, generally cylindrical, smooth walled, slightly curved, 1–3 septate; 1–septate conidia (16.3–) 20.4 (–24.6) × (3.8–) 4.3 (–4.8) µm (*n* = 5), 2–septate conidia (19.6–) 23.5 (–27.3) × (4.2–) 4.8 (–5.7) µm (*n* = 12), 3–septate conidia (22.3–) 26.9 (–31.9) × (3.8–) 4.8 (–5.5) µm (*n* = 37). *Microconidia* and *chlamydospores* not observed on PDA.

Culture characteristics: Colonies after 7 days reached 2–4 cm on PDA, slow-growing with even margin. Colony on PDA is white, mostly reddish-brown in the middle, thick, cottony to felty, areal mycelium, and white or brown hyphal strands partly covered with off-white to cream slime. Colony-reverse outer layer is white to yellowish, and in the middle pigmented with reddish-brown to dark brown.

Material Examined: CHINA, Beijing Province, Shunyi District, Isolated from symptomatic roots (necrotic lesions and black discolorations of the basal end of the root stock and vascular tissues) of *Vitis vinifera*, 15. July. 2019, P.D. Abeywickrama, living cultures JZB 3300001, JZB 3300002.

Note: Morphologically, our two isolates (JZB 3300001 and JZB 3300002) were identical and there were no differences in their DNA sequence data. Thus, we recognize that these two isolates belong to a single species. Phylogenetic analyses of the combined ITS+ *tef1-α*+ *ß-tubulin* multi-locus dataset show that our isolates cluster within the *Campylocarpon* clade sister to ex-type strain of *C. fasicularae* (CBS 112613) with 99% ML support and 1.00 BYPP (Figure 4). Our *C. fasicularae* strains share similar morphology as other *Campylocarpon* asexual morphs [1]. When comparing the morphology with the holo-type strain of *C. fasicularae* (herb. CBS 6590; dried MEA culture of strain CBS 112613), conidiophore and conidial characters showed slight dimensional differences. The conidiophore of our *C. fasicularae* strains are comparatively smaller than those of isolate CBS 112613 (11.9–) 19.1 (–28.7) × (1.9–) 2.9 (–5.5) vs. 15–40 × 6–16). Furthermore, CBS 112613 has relatively larger conidia, while our collection has relatively smaller conidia (see [4] for dimensional data). However, we could not observe the 4-septate or 5-septate conidia in our collection.

### 2.4. Molecular Characterization and Multi-marker Analysis

Sequences from two *Campylocarpon* isolates from the current study and sequences from cylindrocarpon-like species generated in previous studies for the ITS, *tef1–α* and *ß-tubulin* markers were used to construct single-marker (data not shown) and multi-marker phylogenetic trees (Figure 4). These phylogenetic analyses consisted of 69 cylindrocarpon-like isolates obtained from various sources (the majority of these isolates were recovered from diseased grapevine samples), and different geographical locations. All of the isolates from the current study, clustered in a subclade within the *Campylocarpon*/*C. fasciculare* (Figure 3). The combined dataset contained 203 sequences with *N. cinnabarina* (A R 4477) and *N. balansae* (CBS 125119) as the outgroup taxa. Similar multigene phylogenetic trees resulted from both RAxML and Bayesian analyses.

The combined gene analyses comprising 2692 characters including gaps (738 characters for ITS, 1244 characters for *tef1–α* and 702 characters for *ß-tubulin*) and the best RAxML tree with a final likelihood value of -18840.342905, is presented. The matrix had 1284 distinct alignment patterns, with 32.61% undetermined characters. Estimated base frequencies were as follows: A = 0.220829, C = 0.312569, G = 0.231493, T = 0.235109; substitution rates AC = 1.067950, AG = 2.758210, AT = 1.655669, CG = 0.773700, CT = 3.702736, GT = 1.000000; gamma distribution shape parameter α = 1.755404. The final alignment and best tree generated from RAxML analysis is deposited at TreeBASE with submission ID 28460.

In the combined analysis, all 69 isolates grouped into six different clades representing different genera (*Campylocarpon*, *Dactylonectria*, *Ilyonectria*, *Neonectria*, *Thelonectria*) including the outgroup genus *Nectria* (Figure 4). Phylogenetic analyses provided good evidence that two isolates from the current study belonged to *C. fasciculare*, as it formed a sister-clade to the ex-type strain of *C. fasciculare* (CBS 112613), with 99% RAxML bootstrap support and 1.00 Bayesian posterior probability (Figure 4).

### 2.5. Pathogenicity Study

**Figure 5:**
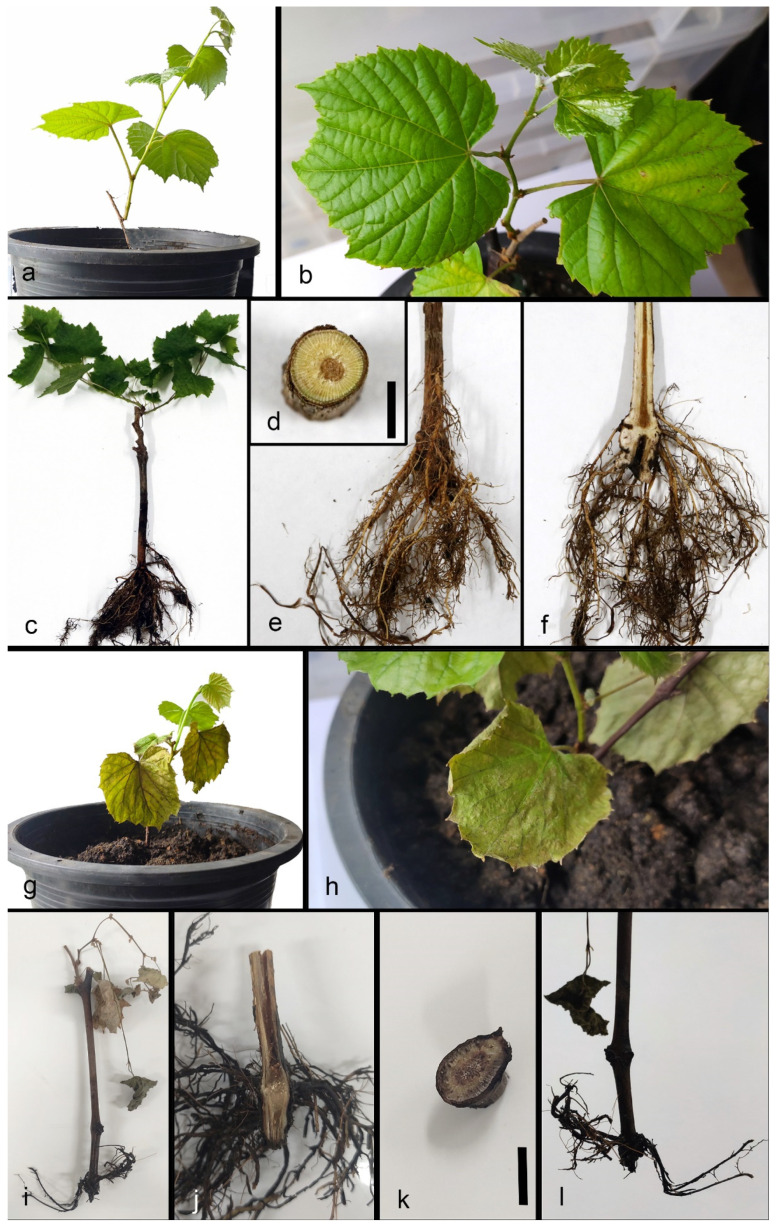
Pathogenicity test of *Campylocarpon fasicularae* in the greenhouse. **a–f** Control plant. **g–l** The plant inoculated with *C. fasicularae.* **g,h** Wilted leaves. **i** Dead vines. **j,k** Black necrosis within the trunk at 3 months post-inoculation. **l** Inoculated plants showed reduced root mass and J root. Scale bar: d, k = 1 cm.

In total, nine young grape plants (six plants for the fungal inoculation and three plants for the control) were used for the pathogenicity study. None of the control plants died or showed any diseased symptoms, while all of the plants inoculated with our isolates died 90 days following inoculation. At 21 days post inoculation (dpi), leaves of three out of six grapevines inoculated with the *Campylocarpon fasicularae* displayed wilted leaves starting from the edge of the leaf blade (Figure 5). At 90 dpi, no discoloration or necrosis was recorded in the control vines, while all six inoculated vines showed a reduced root mass and J root. Analysis of the internal wood symptoms revealed the wood discoloration and necrosis in the stem starting from the base. The pathogen was re-isolated and confirmed as *C. fasicularae*, with 90–100% isolation ratio from dead plants. No *Campylocarpon* species or any fungal species were isolated from any of the control plants (Figure 5).

## 3. Discussion

Grapevine black-foot disease is found in many viticulture regions (Table 1) [27], however, there are no records from China. The current study represents the first attempt to elucidate the incidence of black-foot disease of grapevine in China. This study provides the identification of *Campylocarpon fasicularae*, which is reported for the first time to occur on grapevines in China. This species is known to be the main fungal pathogen associated with black-foot disease of grapevines in Brazil, California, South Africa, Spain, Turkey and the United States (Table 1) [27].

Initially, it was stated that black-foot and Petri diseases were caused by *Phaeomoniella chlamydospora* and *Phaeoacremonium minimum* (≡*Togninia minima*) species [5,13,16]. Due to the resemblance of vascular and foliar symptoms associated with both diseases in the field, disease diagnosis was very difficult based solely on field observations [13]. Later, it was proven that black-foot pathogens were often the part of disease complexes with other fungi or nematodes [16]. Therefore, both Petri and black-foot diseases were responsible for the decline of young grapevine [22]. Currently, it is known that at least 24 cylindrocarpon-like fungal species are associated with black foot disease of grapevine including *Campylocarpon* [27]. *Campylocarpon* species are morphologically similar to *Cylindrocarpon* and *Ilyonectria* species [4,5,16]. However, *Campylocarpon* can be distinguished by having curved macroconidia but no microconidia and chlamydospores [4,5].

### Investigations of Grapevine Trunk Diseases (GTD) in China

Grapevine trunk and root diseases have become one of the main considerable factors that limit both vineyard longevity and productivity in any grape-growing regions. During the past decade, investigations of grapevine trunk and root diseases in Chinese vineyards had increased rapidly and efficiently [28,29,30]. The GTD complex comprises black-foot disease, Botryosphaeria dieback, Esca, Eutypa dieback, Petri disease, and Phomopsis dieback [28,29,30,31,32,33,34,35,36].

In 2013, Yan et al. [29], conducted the first comprehensive study on Botryosphaeria dieback of grapevines in China. They reported four Botryosphaeriaceous taxa causing Botryosphaeria dieback in grapevines; *Botryosphaeria dothidea, Diplodia seriata*, *Lasiodiplodia theobromae* and *Neofusicoccum parvum*, with the support of morpho-molecular and pathological approaches [29].

Later, using advanced molecular techniques such as whole-genome, transcriptome and comparative genome analyses, a group of researchers identified the genomic characteristics of the Botryosphaeriaceous pathogens; *B. dothidea, L. theobromae* and *N. parvum* which were isolated from China [29,30]. In their study, they revealed that, these pathogenic genomes are encoded with a diverse range of genes related to virulence, transporters and the synthesis of secondary metabolites in Botryosphaeriaceae [30]. Further, they have stated, *L. theobromae* utilized different infection strategies to adapt to different lifestyles and these Botryosphaeriaceae species were phylogenetically closer to opportunistic pathogens by sharing common traits with other opportunistic fungal pathogens [30].

Over the last few years, Phomopsis dieback (Diaporthe dieback, Phomopsis cane and leaf spot) has also been recorded in China [28,31]. Currently, ten *Diaporthe* species (*D. eres, D. guangxiensis, D. gulyae, D. hongkongensis, D. hubeiensis, D. pescicola, D. phaseolorum, D. sojae, D. unshiuensis* and *D. viniferae*) are associated with grapevine dieback in Chinese vineyards [28]. Among them, *Diaporthe eres* was identified as the most prominent and widespread species in China [28]. In 2019 using haplotype network analysis, Manawasinghe et al. [28] revealed that the *D. eres* populations from China and Europe have a close relationship in recombination and suggested that the Chinese *D. eres* population may have occurred due to the result of an admixture [28].

Recently, the Esca disease complex was reported in China, and *Phaeoacremonium minimum* was identified as the pathogen, associated with the interveinal leaf necrosis and wood decay symptoms [32]. In early 2021, a basidiomycete fungus; *Fomitiporia punicata* was found to be associated with the Esca complex of grapevine in China and it was confirmed that the presence of *P. minimum* was infected in the same vines with *F. punicata* [33].

Eutypa dieback was first reported in China in 2007 [34]. However, there were not many details available about the incidence of Eutypa dieback in different grapevine cultivars and vineyards. Even though the *Eutypella vitis* was reported as a pathogen in China, it has not been confirmed by performing Koch’s postulates [35].

Several cylindrocarpon-like (*Cylindrocarpon*, *Cylindrocladiella*, *Dactylonectria*, *Ilyonectria*, *Neonectria*) taxa have been recorded in China, from different host plants [36]. Most of these species are known as causing black root rot symptoms to the associated host [36]. Jayawardena et al. [37], was able to obtain operational taxonomic units (OTU) of *Ilyonectria* and *Nectriaceae* species that have been associated with grapevines in China using high-throughput sequencing [37]. However, they were not identified up to species level nor was their pathogenicity confirmed. Therefore, this study is considered as the first comprehensive poly-phasic approach to identify the *Campylocarpon* species associated with black-foot disease of grapevine in Chinese vineyards.

During this study, we observed fungal hyphae on uprooted diseased vine rootstocks (Figure 1 and Figure 2). Furthermore, dark brown-black discoloration has also been found on the wood in the buried trunk portion. Similar observations have been made by Sweetingham [38], and he suggested that this may be because of infection occurred at a later stage in a vineyard [2,39]. However, we could not investigate planting materials from the nursery or analyze the soil from the field. Hence, the initial source of *C. fasciculare* infection, is still undetermined.

We have observed numerous external symptoms on young grapevines [see result Section 2.1]. Grape growers have experienced these field symptoms on young vines shortly after transplantation (personal communication). Disease symptoms have been noticed early in the growing season as affected vines showed poor new growth, failed to form new shoots, reduced vegetative growth and, some vines even died. Researchers stated that environmental factors and host stress conditions increased the black-foot disease development in vineyards [2]. Poor soil preparation and soil compaction may have resulted in poor root development in young grapevines (J rooting) [2]. High-temperature conditions during the summer season also played a vital role in disease symptom development in vineyards [2]. It was stated that, during the last decade, northern China has become significantly warmer [28]. Therefore, we suspected that human-mediated factors or/and adverse environmental factors may influence the emergence of novel pathogens such as *C. fasciculare* in Chinese vineyards.

## 4. Materials and Methods

### 4.1. Field Surveys and Fungal Isolation

During the survey of grapevine trunk and root disease causing pathogens in China, in July 2019, symptomatic young grapevines were collected from a vineyard in Shunyi District (40°09′00″ N–116°39′41″ E), Beijing province of China (Figure 1 and Figure 2). Five randomly selected diseased young grapevine plants were uprooted and samples were taken to the laboratory for further observations and isolations.

Diseased plants were washed with running tap water to remove debris and soil particles and air dried to remove excess water. Isolates were recovered from the basal end of the root stock and necrotic roots, that exhibited symptoms of black-foot. Tissues cut from both affected and healthy areas (each about 5 mm^2^), were surface sterilized by 1.5% sodium hypochlorite solution (NaOCl) for 1 min., and washed with sterilized distilled water. Then tissues were soaked in 75% ethyl alcohol for 1 min., and rinsed three times in sterilized distilled water. Tissue pieces were plated on potato dextrose agar (PDA; Oxoid) supplemented with Ampicillin (100 µL/mL, Sigma-Aldrich (Shanghai) Trading Co. Ltd., Shanghai, China). PDA plates (90 mm diam.) were incubated 5–10 days at 25 ± 2 °C with a 12-h photoperiod. Single hyphal tip and single spore isolations were carried out to obtain pure cultures. PDA slants for purified culture were prepared and saved at 4 °C.

### 4.2. Morphological Characterization

All fungal colonies were grown at 25 ± 2 °C with a 12-h photoperiod for 5–10 days. Macroscopic characters such as colony texture, color and the type of growing margin were evaluated on PDA. Colonies grown on PDA were further incubated up to 2 weeks to determine the microscopic characters. The fungal structures were mounted in water for microscopic studies and photographed with a Zeiss Axio Imager Z2 microscope (Carl Zeiss Microscopy, Oberkochen, Germany).

### 4.3. Molecular Characterization and Multi-Marker Analysis

Total genomic DNA was extracted from purified fungal colonies using cetyltrimethylammonium bromide (CTAB) extraction method followed by Jeewon et al. [40] and Suwannarach et al. [41] with some modifications. Approximately 0.05 g of mycelium was mixed with liquid nitrogen and ground into a fine powder with a handheld tissue homogenizer (1800/8000RPM Mini Grinding Mill MY-10/20, Jingxin Technology, China). The powdered mycelium was suspended in 500 µL of pre-heated CTAB (2% (*w/v*) CTAB; 100 mM Tris-HCl; 1.4 M NaCl; 20 mM EDTA, pH 8.0] and incubated for 1 hr at 65 °C. Then 250 µL of Tris-HCl and 250 µL of chloroform: isoamyl alcohol (24:1, *v/v*) was added, thoroughly mixed, and the tube was centrifuged for 10 min at 10,000 rpm. The extraction was repeated until the interface was clear. The supernatant was moved to a new Eppendorf tube, added 0.6 volumes of Isopropyl alcohol and kept in room temperature for 1 hr. Later, the tube was centrifuged for 10 min at 12,000 rpm and the upper layer was discarded. After DNA precipitation, the pellet was washed with 70% ethanol and dried at room temperature. It was resuspended in 20–30 µL of 0.002% RNase (5 mg/mL) in TE buffer and incubated for 1 h at 37 °C. Extracted DNA was stored at –20 °C for further analyses.

The ITS, *tef1–α* and *ß-tubulin* loci were amplified; respective primers used and protocols are summarized in Suppl. Table S1. Polymerase chain reaction (PCR) was carried out in a volume of 25 μL, which contained 12.5 µL of 2 × Taq PCR Master-Mix (Biomed Co., China), 1 μL of each primer (forward and reversed), 1 μL genomic DNA, and 9.5 μL of deionized water. PCR amplification was confirmed on 1% agarose electrophoresis gels stained with ethidium bromide and visualized under UV light using Gel Doc XR + Molecular Imager Imaging system (BIO-RAD, USA). The amplified PCR fragments were sent to a commercial sequencing provider and sequenced by the Biomed Company, Beijing, China. The nucleotide sequence data obtained was deposited in GenBank; their accession numbers are given in Suppl. Table S2.

#### Phylogenetic Analysis

Generated sequences were assembled to obtain consensus sequences with DNAMAN 6.0 (Lynnon Biosoft, Foster City, CA, USA).

Sequences with high similarity with the new taxon were determined by using the NCBI (National Center for Biotechnology Information) Nucleotide BLAST (Basic Local Alignment Search Tool) search engine (https://blast.ncbi.nlm.nih.gov/, accessed on 8 August 2021). Additional reference sequences obtained from GenBank and a previous study [24], are listed in Suppl. Table S2.

Individual data sets for each gene region were aligned using the multiple alignment program for amino acid or nucleotide sequences (MAFFT) version 7 (https://mafft.cbrc.jp/alignment/software/, accessed on 8 August 2021) [42], and manual improvements were done where necessary by using BioEdit sequence alignment editor software version 7.0.9.0 [43]. Aligned gene regions were combined using BioEdit version 7.0.9.0, and the final alignment consists of three gene regions with the following gene order: ITS, *tef 1–α* and *ß-tubulin*. FASTA formats were changed to PHYLIP and NEXUS formats by using ALTER (Alignment Transformation Environment) (http://www.sing-group.org/ALTER/, accessed on 8 August 2021). Phylogenetic analyses of combined markers were performed using maximum likelihood (ML), and Bayesian Inference (BI) criteria.

Best models for BI and ML were selected independently for each locus using MrModeltest v. 3.7 [44] under the Akaike Information Criterion (AIC) implemented in PAUP v. 4.0b10. ML trees were generated using RAxML-HPC2 on XSEDE (8.2.8) [45,46] in the CIPRES Science Gateway platform. GTR + I + G were used as the model of evolution and Bootstrap support obtained by running 1000 pseudo replicates.

Bayesian Inference (BI) analysis was conducted with MrBayes v. 3.1.2 to evaluate posterior probabilities (BYPP) [47,48] by Markov Chain Monte Carlo sampling (BMCMC). Two parallel runs were conducted, using the default settings, but with the following adjustments: Six simultaneous Markov chains were run for 2,000,000 generations and trees were sampled every 1000th generation. The first 25% of generated trees were discarded and the remaining 75% of trees were used to calculate posterior probabilities (PP) of the majority rule consensus tree. Phylograms were visualized with the Fig Tree v1.4.0 program [49] and reorganized using Microsoft Office PowerPoint 2010 and Adobe Illustrator CS5 (Version 15.0.0, Adobe, San Jose, CA). ML bootstrap supports (greater than or equal to 60%) and Bayesian posterior probabilities (greater than or equal to 0.90%) are shown at the nodes. Final alignment and phylogenetic tree were submitted to the TreeBASE (https://treebase.org/, accessed on 8 August 2021).

### 4.4. Pathogenicity Study

#### 4.4.1. Inoculum Preparation

In order to assess the pathogenicity, mycelium of the isolates (JZB 3300001 and JZB 3300001) was produced on sterilized sorghum grains. Two hundred grams of sorghum grains were placed in 500 mL conical flasks containing 300 mL of water and heated to boiling temperature (each isolate in three flasks). The grains were left to settle for 20 min, then washed three times with running tap water and drained off the excess water. The boiled grains were autoclaved at 121 °C for 15 min at 15 psi on each day, for two consecutive days. The sorghum grains were inoculated with five mycelium plugs, 7 mm in diameter, taken from the margins of cultures. The flasks were incubated at 25 ± 2 °C in a shaking incubator at the speed 150 rpm for 14 days. During the incubation time sorghum grains were visually assessed as being well colonized by mycelium. Furthermore, flasks were shaken daily for 3–5 s. by hand to facilitate the even colonization more. Control sorghum grains were left uncolonized.

#### 4.4.2. Plant Material Preparation

Plants of grapevine variety SB (*Vitis vinifera* cv. Summer Black) was used for the pathogenicity experiment. Two node cuttings were grown to rooted plants used for the inoculation. To obtain the rooted plants, the cuttings were potted in compost soil + coconut coir trays, in a plant house at 25 °C for 1 month. The rooted plants were carefully uprooted from the trays and washed with running tap water to remove the debris before inoculation.

#### 4.4.3. Pot Preparation and Inoculation

The 1.5 L pots were ¾ filled with potting mixture, containing compost, soil and coconut coir. Each potting mix was well mixed with colonized or uncolonized sorghum grains obtained from inoculum preparation. A 5–6 cm hollow was made and rooted plants were placed in the hole and the pot was filled. Three plant replicates for each isolate were arranged and three plants were kept as control. The pots were laid on the bench in a greenhouse with 12-h/day light in 25–30 °C temperature (12-h/day dark and 23–25 °C temperature at night). All plants were lightly watered daily.

Disease development and symptoms were evaluated by counting dead plants for three months. Koch’s postulates were confirmed by making isolations from the plants to confirm the continuing presence of the inoculated fungus. The lower stem sections and roots were used to re-isolate the pathogens from the infected plants (method mentioned above). Fungal isolates grown out from the woody pieces were identified by comparing colony morphology and conidial characters with the cultures of the *Campylocarpon fasciculare* isolates that were used to inoculate the plants. Furthermore, re-isolated fungal strains were subjected to sequencing of the ITS region to confirm their identity as *C. fasciculare*. The experiment was repeated once.

## 5. Conclusions and Future Prospects

The black-foot disease of grapevine is a completely new and as yet poorly documented disease that affect grapevines in China. The diverse fungal species are associated with the disease worldwide and here we reported *C. fasciculare* for the first time from China. Black-foot causing pathogens including *C. fasciculare*, can infect the grapevines in both nursery and young vineyard stages. Even though no cure is known for the grapevine black-foot disease, integrated disease management practices are recommended. To manage the black-foot disease, grape growers are advised to control human-mediated and environmental stress conditions in vineyards, especially soil compaction, poor drainage and high temperature. Extensive field surveys, sample collections throughout China and utilizing advanced molecular techniques such as next-generation sequencing are recommended for further studies. The results presented in this study provide inputs for several fields, including disease-resistant cultivar breeding, fungicides screening, and developing appropriate quarantine and management strategies for prevention and control of black-foot disease and other GTD’s.

## Figures and Tables

**Table 1:** Previously recorded species associated with black-foot disease on grapevines (*Vitis* spp. or *Vitis vinifera*) and their geographical distribution (https://nt.ars-grin.gov/fungaldatabases/fungushost/FungusHost; accessed on 8 August 2021.).

Genus	Species	Geographical Distribution
* Campylocarpon *	* C. fasciculare *	Brazil, South Africa, Spain, Turkey, United States
	* C. pseudofasciculare *	Brazil, Peru, South Africa, Uruguay
* Cylindrocarpon *	* C. cylindroides *	Canada, United States
	*C. destructans—*(*Ilyonectria destructans*)	Argentina, Canada, France, Iran, South Africa, Spain
	*C. destructans var. crassum—*(*Ilyonectria crassa*)	Uruguay
	*C. destructans var. destructans—*(*Ilyonectria destructans*)	Canada
	*C. liriodendri—*(*Ilyonectria liriodendra*)	Australia, Canada, France, Iran, Portugal, Spain, Switzerland, Uruguay, United States
	*C. macrodidymum—*(*Dactylonectria macrodidyma*)	Australia, Canada, Chile, New Zealand, Slovenia, South Africa, Spain, Turkey, Uruguay, United States
	*C. obtusisporum—*(*Neonectria obtusispora*)	United States
	*C. olidum—*(*Thelonectria olida*)	Uruguay
	*C. olidum var. crassum*	Uruguay
	*C. pauciseptatum—*(*Dactylonectria pauciseptata*)	Canada, New Zealand, Portugal, Slovenia, Spain, Uruguay
	*Cylindrocarpon* spp.	Australia, Canada, Florida, Lebanon, New Zealand, Portugal, South Africa, Spain, Switzerland, Tasmania, United States
	* C. tonkinense *	India
* Cylindrocladiella *	* C. lageniformis *	South Africa, United States
	* C. parva *	New Zealand, South Africa, Spain
	* C. peruviana *	Peru, South Africa, Spain, United States
	* C. pseudoparva *	New Zealand
	*Cylindrocladiella* spp.	New Zealand
	* C. viticola *	South Africa
	* C. vitis *	New Zealand
* Dactylonectria *	* D. alcacerensis *	Portugal, South Africa, Spain, United States
	* D. estremocensis *	Portugal
	* D. hordeicola *	France
	* D. macrodidyma *	France, Portugal, South Africa, Spain, United States
	* D. novozelandica *	New Zealand, South Africa, United States
	* D. pauciseptata *	Bulgaria, France, Slovenia, South Africa, Spain
	* D. pinicola *	Portugal
	* D. torresensis *	Czech Republic, France, Italy, New Zealand, Portugal, South Africa, Spain
	* D. vitis *	Portugal
* Ilyonectria *	* I. europaea *	Portugal
	* I. liriodendri *	Australia, Brazil, Canada, France, Iran, Italy, New Zealand, Portugal, South Africa, Spain, Switzerland, Turkey, United States, Uruguay
	* I. lusitanica *	Portugal
	* I. pseudodestructans *	Portugal
	* I. robusta *	Brazil, British Columbia, Canada, France, Portugal, Spain, United States
	* Ilyonectria sp *	Australia, Portugal
* Neonectria *	* N. macrodidymum *	Canada, South Africa, United States
	* N. mammoidea *	United States
	* N. microconidia *	Japan
	* N. radicola *	Canada
* Thelonectria *	* T. aurea *	United States
	* T. blackeriella *	Italy

## Supplementary Materials

The following are available online at https://www.mdpi.com/article/10.3390/pathogens10121555/s1, Table S1: Gene regions, respective primers, PCR protocols and references used in the study, Table S2: Strains/isolates and their accession numbers used in phylogenetic analyses.
