# Supplementary material for: Campylocarpon fasciculare (Nectriaceae, Sordariomycetes); Novel Emergence of Black-Foot Causing Pathogen on Young Grapevines in China
Source: Pathogens. 2021 Nov 29;10(12):1555. doi: 10.3390/pathogens10121555 (PMC8708891; doi:10.3390/pathogens10121555)
Supplement: Supplementary file 1 [file pathogens-10-01555-s001.zip › pathogens-1462367-supplementary.pdf]

Table S1. Gene regions, respective primers, PCR protocols and references used in the study.

Gene	Primers	PCR conditions:(Annealing temp. in bold)	References
ITS	ITS5<br>ITS4	(95 °C: 30 s, **59** °C:50 s, 72 °C: 90 s) × 35 cycles	White et al. (1990)
tef1–α	728F<br>986R	(94 ˚C: 30 s, **54** ˚C: 30 s, 72 ˚C: 30 s) × 35 cycles	Carbone and Kohn (1999)
ß-tubulin	T1<br>Bt 2b	(94 ˚C: 6 min, **56** ˚C: 60 s, 72 ˚C: 45 s) × 35 cycles	O'Donnell and Cigelnik (1997)<br>Glass and Donaldson (1995)

Table S2. Strains/isolates and their accession numbers used in phylogenetic analyses.

Species	Isolate	ITS	*tef1-α*	*ß-tubulin*
Campylocarpon fasciculare	CBS 112613	AY677301	JF735691	AY677221
C. fasciculare	KARE1889	MK400278	MK409922	MK409845
C. fasciculare	KARE1890	MK400279	MK409923	MK409846
C. fasciculare	KARE1891	MK400280	MK409924	MK409847
C. fasciculare	KARE1892	MK400281	MK409925	MK409848
C. fasciculare	KARE1893	MK400282	MK409926	MK409849
C. fasciculare	KARE1894	MK400283	MK409927	MK409850
C. fasciculare	KARE1895	MK400284	MK409928	MK409851
C. fasciculare	**JZB 3300001**	**OK605034**	**OK595364**	**OK595362**
C. fasciculare	**JZB 3300002**	**OK605035**	**OK595365**	**OK595363**
C. pseudofasciculare	CBS 112679	AY677306	JF735692	AY677214
Dactylonectria alcacerensis	CBS 129087	JF735333	JF735819	AM419111
D. alcacerensis	Cy133	JF735331	JF735817	JF735459
D. alcacerensis	KARE413	MK400304	MK409948	MK409871
D. amazonica	MUCL55430	MF683706	MF683664	MF683643
D.amazonica	MUCL55433	MF683707	MF683665	MF683644
D. anthuriicola	CBS 129085	JF735302	JF735768	JF735430
D. ecuadoriensis	MUCL55424	MF683704	MF683662	MF683641
D. ecuadoriensis	KARE2108	MK400316	MK409960	MK409883
D. estremocencis	CBS 129085	JF735320	JF735806	JF735448
D. estremocencis	CPC 13539	JF735330	JF735816	JF735458
D. hispanica	CBS 142827	KY676882	KY676870	KY676876
D. hispanica	Cy228	JF735301	JF735767	JF735429
D. macrodidyma	CBS 112615	AY677284	JF735833	AY677229
D. macrodidyma	Cy123	JF735341	JF735837	JF735470
D. macrodidyma	Cy139	AM419071	JF735839	AM419106
D. macrodidyma	KARE423	MK400300	MK409944	MK409867
D. novozelandica	CBS 113552	JF735334	JF735822	AY677237

D. novozelandica	Cy115	JF735335	JF735823	JF735460
D. polyphaga	MUCL55209	MF683689	MF683647	MF683626
D. polyphaga	MUCL54802	MF683698	MF683656	MF683635
D. torresensis	CBS 129086	JF735362	JF735870	JF735492
D. torresensis	Cy118	JF735354	JF735859	JF735483
D. valentina	CBS 142826	KY676881	KY676869	KY676875
D. valentina	KARE2111	MK400314	MK409958	MK409881
D. vitis	CBS 129082	JF735303	JF735769	JF735431
Ilyonectria capensis	CBS 132815	JX231151	JX231119	JX231103
I. capensis	KARE1920	MK400330	MK409974	MK409897
I. crassa	CBS 139.30	JF735275	JF735723	JF735393
I. destuctans	CBS 264.65	AY677273	JF735695	AY677256
I. europaea	CBS 129078	JF735294	JF735756	JF735421
I. liriodendri	CBS 110.81	DQ178163	JF735696	DQ178170
I. mors-panacis	CBS 306.35	JF735288	JF735746	JF735414
I. palmarum	CBS 135754	HF937431	HF922614	HF922608
I. robusta	CBS 308.35	JF735264	JF735707	JF735377
I. venezuelensis	CBS 102032	AM419059	JF735760	AY677255
Nectria balansae	CBS 125119	HM484857	HM484848	HM484874
N. cinnabarina	A.R. 4477	HM484548	HM484527	HM484606
Neonectria californica	KARE1838/CBS145774	MK400332	MK409976	MK409899
N. ditissima	CBS 226.31	JF735309	JF735783	DQ789869
N. lugdunensis	CBS 125485	KM231762	KM231887	KM232019
N. major	CBS 240.29	JF735308	JF735782	DQ789872
N. neomacrospora	CBS 324.61	JF735312	HM364352	DQ789875
N. obtusispora	CBS 183.36	AM419061	JF735796	AM419085
N. ramulariae	CBS 151.29	JF735313	JF735791	JF735438
Thelonectria acrotyla	G.J.S. 90-171	JQ403329	JQ394751	JQ394720
T. amamiensis	MAFF 239819	JQ403337	KJ022348	JQ394727
T. aurea	KARE1830/CBS 145584	MK400285	MK409929	MK409852
T. blackeriella	BF142	KX778711	-	KX778702
T. diademata	A.R. 4765	NR137784	JQ394736	JQ394700
T. gongylodes	G.J.S. 04-171	JQ403318	JQ394744	JQ394710
T. nodosa	G.J.S. 04-155	JQ403317	JQ394743	JQ394709
T. olida	CBS 215.67	KJ021982	-	KM232024
T. stemmata	C.T.R. 71-19	JQ403312	JQ394739	JQ394704
T. torulosa	A.R. 4764	JQ403309	KJ022389	JQ394701
T. trachosa	CBS 112467	KF569842	KF569860	KF569869
T. truncata	G.J.S. 04-357	JQ403319	JQ394745	KJ022324
T. truncata	MAFF241521	JQ403339	KJ022325	JQ394757
T. veuillotiana	G.J.S. 92-24	JQ403335	JQ394755	JQ394725
